# Prognostic Factors of 1-Year Postoperative Functional Outcomes of Older Patients with Intertrochanteric Fractures in Thailand: A Retrospective Cohort Study

**DOI:** 10.3390/ijerph18136896

**Published:** 2021-06-27

**Authors:** Nath Adulkasem, Phichayut Phinyo, Jiraporn Khorana, Dumnoensun Pruksakorn, Theerachai Apivatthakakul

**Affiliations:** 1 Department of Orthopedics, Faculty of Medicine, Chiang Mai University, Chiang Mai 50200, Thailand; adulkasem.n@gmail.com (N.A.); dumnoensun@hotmail.com (D.P.); tapivath@gmail.com (T.A.); 2 Center for Clinical Epidemiology and Clinical Statistics, Faculty of Medicine, Chiang Mai University, Chiang Mai 50200, Thailand; jiraporn.kho@elearning.cmu.ac.th; 3 Department of Family Medicine, Faculty of Medicine, Chiang Mai University, Chiang Mai 50200, Thailand; 4 Musculoskeletal Science and Translational Research (MSTR) Cluster, Chiang Mai University, Chiang Mai 50200, Thailand; 5 Division of Pediatric Surgery, Department of Surgery, Faculty of Medicine, Chiang Mai University, Chiang Mai 50200, Thailand

**Keywords:** fracture fixation, geriatric, intertrochanteric fractures, prognostic factors, Thai

## Abstract

Restoration of ambulatory status is considered a primary treatment goal for older patients with intertrochanteric fractures. Several surgical-related parameters were reported to be associated with mechanical failure without focusing on the functional outcomes. Our study examines the roles of both clinical and surgical parameters as prognostic factors on 1-year postoperative ambulatory outcomes, reaching a good functional outcome (the New Mobility Score: NMS ≥ 5) and returning to preinjury functional status at one year, of older patients with intertrochanteric fracture. Intertrochanteric fractures patients age ≥65 years who underwent surgical treatment at our institute between January 2017 and February 2020 were included. Of 209 patients included, 149 (71.3%) showed a good functional outcome at one year. The pre-injury ambulatory status (OR 52.72, 95%CI 5.19–535.77, *p* = 0.001), BMI <23 kg/m^2^ (OR 3.14, 95%CI 1.21–8.13, *p* = 0.018), Hb ≥10 g/dL (OR 3.26, 95%CI 1.11–9.57, *p* = 0.031), and NMS at discharge ≥2 (OR 8.50, 95%CI 3.33–21.70, *p* < 0.001) were identified as independent predictors for reaching a good postoperative functional outcome. Only aged ≤80 (OR 2.34, 95%CI 1.11–4.93, *p* = 0.025) and NMS at discharge ≥2 (OR 6.27, 95%CI 2.75–14.32, *p* < 0.001) were significantly associated with an ability to return to preinjury function. To improve postoperative ambulatory status, orthopedic surgeons should focus more on modifying factors, such as maintaining the preoperative hemoglobin ≥10 g/dL and providing adequate postoperative ambulation training to maximize the patients’ capability upon discharge. While surgical parameters were not identified as predictors, they can still be used as guidance to optimize the operation quality.

## 1. Introduction

Intertrochanteric fracture is one of the most common fractures in the geriatric population and is associated with serious consequences [1]. Even though the chance of uneventful bony healing was high, only half of the patients or less could return to their preinjury ambulatory level after operation [2]. The lack of self-ambulatory capability leads to poor quality of life after the injury and subsequently causes significant medical and socioeconomic burdens to both the patients and their families [3]. Therefore, the ultimate treatment goal in treating this fracture type is to enable the patients to return to their previous functional status and social participation [2,4]. As non-operative management was proven to be associated with high morbidity and mortality, operative treatment is currently regarded as the gold standard for the treatment of intertrochanteric fracture [5,6]. 

Conventionally, the anatomical reduction of the fracture was expected to restore the pre-injury alignment of the patient [7]. Anatomical to slightly valgus femoral neck-shaft angle was generally accepted as an adequate coronal alignment of the proximal femur [8]. Displacement of the femoral cortical support both in the coronal and the sagittal plane should be reduced. Recently, extramedullary reduction was found to have good mechanical properties compared to intramedullary reduction [9]. However, the position of the proximal fixation in three dimensions still greatly affected the fixation stability whether the intramedullary or extramedullary implant was used. 

Several parameters were proposed to determine the quality of surgical fixation, such as the tip apex distance (TAD) and its variation calcar reference TAD (CalTAD), which determines the distance of the implant and screw position of proximal fixation in the coronal and axial plane [7,10]. Another important parameter is Parker’s ratio, which was used to assess the supero-inferior position of the fixation in the proximal femur [11]. These surgical parameters were widely studied and have proven to be predictive of mechanical failure [7,10], which was the mediator on the causal pathway to the final ambulatory status of the patients [12]. Therefore, it might be reasonable to hypothesize that these surgical-related factors could also have the potential to predict the postoperative functional outcome of the patients.

Several prognostic factors for functional outcomes of patients with intertrochanteric fracture after their surgical treatment were reported in the literature [4,13]. However, most of the evidence tends to focus on clinical parameters and patients’ baseline conditions. A previous systematic review of 33 studies identified anemia on admission, comorbidity, pre-fracture function, and cognitive impairment as predictors of postoperative ambulatory status [14]. There was little evidence supporting the association of surgical-related factors with postoperative ambulatory capability. Our study aims to examine the roles of both clinical and surgical parameters as prognostic factors for 1-year postoperative functional outcomes of older patients with intertrochanteric fracture. Two functional outcomes were explored in this study, namely, an ability to reach a good postoperative ambulatory status and an ability to return to preinjury ambulatory status at one year.

## 2. Materials and Methods

### 2.1. Design and Setting

Prognostic factor research was conducted with a retrospective observational cohort design. We included patients with intertrochanteric fractures who underwent a surgical operation at Maharaj Nakorn Chiang Mai hospital from January 2017 to February 2020. Our institute is a university-affiliated, tertiary care medical center responsible for the specialized care of patients within the upper Northern region of Thailand. This study was approved by the Research Ethics committee of the Faculty of Medicine, Chiang Mai University (No. 101/2021 Study code ORT-2564-07985).

### 2.2. Study Patient

We defined the study domain as older patients with an intertrochanteric fracture who underwent surgical management. The medical records of the patients were retrieved and reviewed based on the International Classification of Diseases, 10th revision, Clinical Modification (ICD-10-CM) Diagnosis Code S72.14 Intertrochanteric fracture of the femur. During the study period, all patients aged more than 65 diagnosed with fragility intertrochanteric fracture, which was caused by low energy trauma and treated with internal fixation, were included. Patients whose fractures were caused by a high mechanism of injury, including polytraumatized patients, patients with previously injured ipsilateral hip or major injury affecting lower extremities deformity, or patients diagnosed with a pathological fracture, were excluded. Patients who could not be reached for the telephone interview or who were unable to provide the data (e.g., passed away) were also excluded.

### 2.3. Treatment Protocol

All patients with intertrochanteric fractures scheduled for operation at our institution are provided with preoperative evaluation by anesthesiologists and medical consultations if required. Closed reduction and internal fixation is generally planned to be performed within the first 72 h after admission. However, if the patients were clinically unstable or deemed unfit for operation, the time to operation would be prolonged. While waiting for the operation, all patients are immobilized with weighted skin traction (2 kg). Board-certified orthopedic trauma surgeons operate using standard surgical techniques [15]. The choice of fixation implants depends on the operating surgeons. For the intramedullary device, Proximal Femoral Nail Antirotation (PFNA) (Synthes, Oberdorf, Switzerland) w used. For extramedullary device, a dynamic hip screw (Synthes, Oberdorf, Switzerland) is used. Postoperative radiography is performed in all cases. 

After the operation, all patients are managed according to the standard protocol, including pain management and mechanical venous thromboembolism prophylaxis. Early rehabilitation, including a range of motion and walker-assisted weight-bearing exercises, is performed as tolerated. Patients are evaluated daily by attending orthopedic surgeons. Shared decision making is used to decide appropriate timing for hospital discharge. Patients were scheduled for clinical and radiographic evaluation at two weeks, four weeks, three months, six months, and one year after the operation.

### 2.4. Data Collection

The data on demographic data (i.e., age at the time of injury and gender) and clinical characteristics (i.e., Charlson’s comorbidity index (CCI) [16] and time to surgery) were retrieved from electronic medical records. Body mass index (BMI) was calculated using weight and height, which were preoperatively estimated and recorded by experienced anesthesiologists. Preoperative laboratory investigations, such as hemoglobin level (Hb), and albumin level (Alb), were also collected. Fracture configuration was classified according to AO/OTA classification [17]. The lateral wall thickness was measured according to the methods described in Gotfried’s study [18]. 

Reduction and fixation parameters assessment were measured from the immediate post-operative radiography. Neck shaft angle and Medial and anterior cortical displacement were measured to identify the post-reduction alignment. Fixation parameters, including CalTAD and Parker’s ratio, were recorded [10]. Implants were classified into either intramedullary or extramedullary devices. 

For avoiding violating the linearity assumption during modeling and interpretability of results, all continuous data were categorized using previously reported cut off points: age less than 80 years [19], preinjury new mobility score (NMS) more than 4 [20], Hb at least 10 g/dL [21], CCI below 5 [16], albumin not lower than 3 mg/L, the lateral wall thickness at least 20.5 mm [18], the neck-shaft angle at least 130° [7], lateral displacement less than 6 mm, posterior displacement less than 7 mm [7,9], CalTAD less than 25 mm [7], Parker’s ratio less than 40% [10], time to surgery less than 3 days [22], hospital stay less than 2 weeks [23]. A good ambulatory status at the discharge time was categorized into two groups based on the consensus of two senior authors (TA and DP): those who could walk with or without gait aids (an NMS of at least 2) and those who could not [24].

### 2.5. Study Endpoint

Postoperative functional outcome at one year was evaluated with the new mobility score (NMS). NMS is comprised of three comprehensive categories describing ambulation ability. The level of dependence was described according to the ability to ambulate with or without aids in the following circumstances: within the house, out of the house, or shopping. The maximum score added up to 9 [20]. This score was widely used and validated with high inter and intra-observer reliability in predicting long-term mortality and rehabilitation outcome in patients with hip fractures [25]. In this study, preinjury NMS, NMS at time of discharge, as well as 1-year postoperative NMS were investigated via structured telephone interviews by investigators who were blinded to the patients’ clinical information to avoid interviewer bias [26]. 

The primary endpoint of this study was the ability to reach a good function outcome at 1-year. According to Parker et al., an acceptable functional outcome was defined as the NMS at least 5 [20]. Other than the ability to reach a good functional outcome at 1-year, another clinically significant endpoint worth exploring was the ability of the patients to return to preinjury functional status at 1-year after the operation, which was set as a secondary endpoint to be explored.

### 2.6. Statistical Analysis

Based on standard recommendations, a minimum of 10 interested endpoints per predictor is required [27]. Thus, an expected number of 180 patients with an intertrochanteric fracture with good postoperative ambulatory status is required to model 18 predictors. Data distribution was tested using histogram and Shapiro–Wilk test. Normally distributed continuous data were presented with means and standard deviation (SD). In contrast, non-normally distributed data were presented with median and interquartile range (IQR), which were tested using t-test and Mann–Whitney U test, respectively. Categorical data were presented in proportion and compared using Fisher’s exact probability test. 

Univariable logistic regression analysis was performed to evaluate the individual effect size (univariable odds ratio, uOR) and the statistical significance of each parameter. The dependent variables for the primary and secondary endpoint were NMS ≥ 5 and returning to preinjury functional status, respectively. Since this study was an exploratory prognostic factors research, we employed a full model approach where all predictors were entered in multivariable logistic regression modelling without stepwise selection or backward elimination. The mode results were presented with multivariable odds ratios (mOR) and their corresponding 95% confidential intervals (CI). All statistical analysis was computed using Stata 16 (StataCorp LLC, College Station, TX, USA). The level of statistical significance was set at *p* < 0.05. 

## 3. Results

**Figure 1:**
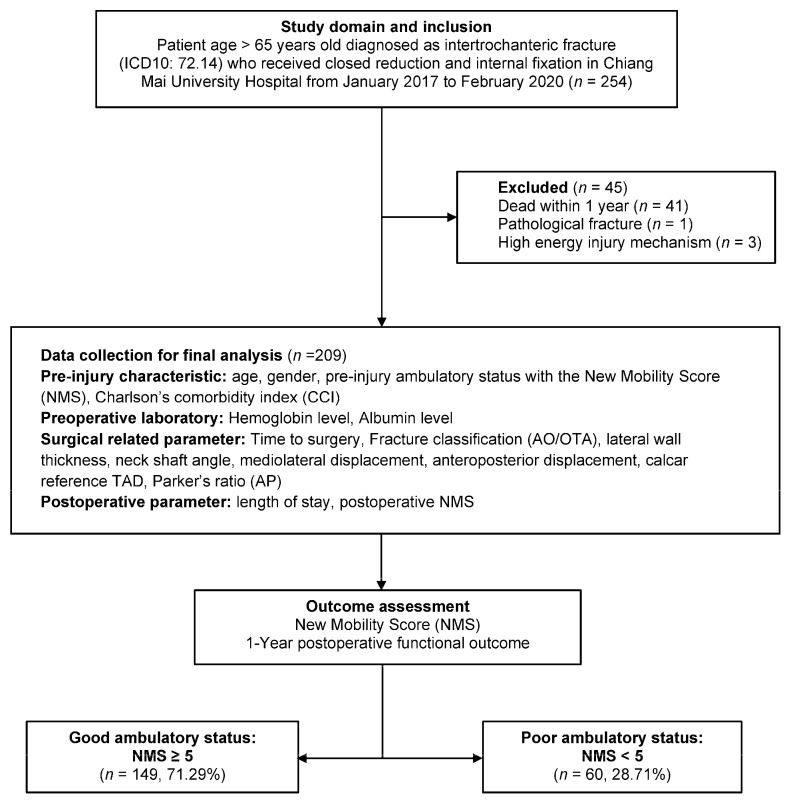
Study flow diagram of eligible patient with fragility intertrochanteric fracture who received reduction and internal fixation. Patient with available 1-year postoperative ambulatory status information were included from January 2017 to February 2020.

There were 254 patients with an intertrochanteric fracture who underwent surgical treatment during the study period at our institution. Among them, 41 patients who passed away before the interview, three polytraumatized patients, and one patient with pathological fracture were excluded. In summary, a total of 209 patients were included in the analysis (Figure 1).

Of the 209 patients, 149 (71.3%) had good postoperative ambulatory status at one year (i.e., NMS ≥ 5). However, only 57 (26.5%) were able to return to preinjury functional status (50 (25%) and 4 (23.5%) in patients with good and poor preinjury ambulatory status respectively). Patients with good postoperative functional outcome had significantly younger age (81 ± 7 vs. 84 ± 6 years, *p* = 0.008), better pre-injury NMS (9 (IQR 7, 9) vs. 6 (IQR 4, 9), *p* < 0.001), higher hemoglobin level (10.7 ± 1.6 vs. 10.2 ± 1.7 g/dL, *p* = 0.022), lower CCI (4 (IQR 4, 5) vs. 5 (IQR 4, 6), *p* < 0.001), lower length of stay (11 (IQR 8, 14) vs. 14 (IQR 11, 18) days, *p* < 0.001), and better NMS at discharge time (3 (IQR 2, 5) vs. 1 (IQR 0, 2), *p* < 0.001) (Table 1). None of the surgical-related factors showed statistically significant differences between the two groups (Table 2).

**Figure 2:**
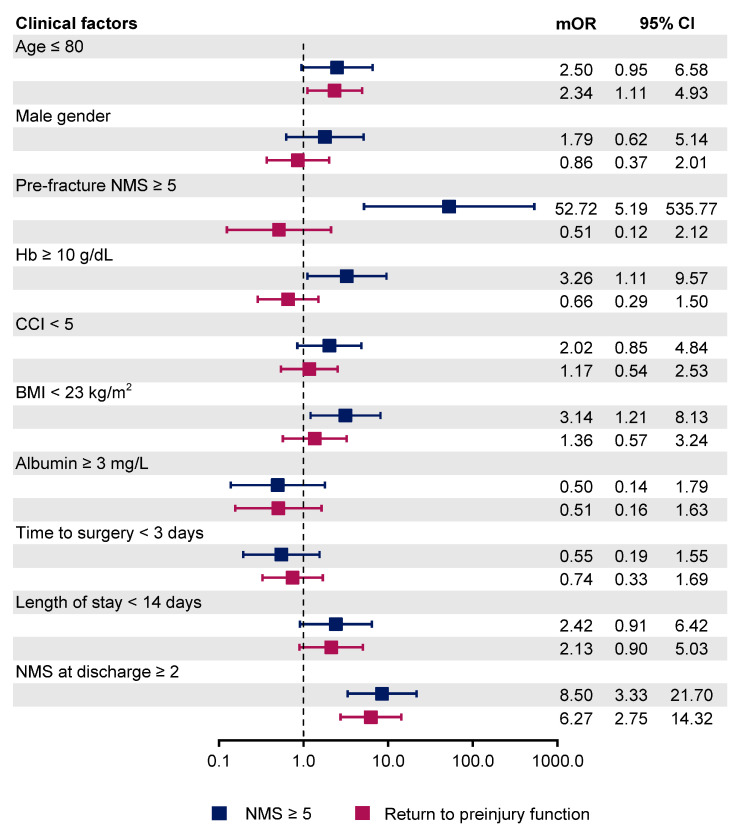
Coefficient plot presenting the odds ratio of each clinical parameter regarding two different endpoints (NMS ≥ 5 and the ability to return to preinjury functional status). Hb: Hemoglobin, CCI: Charlson’s Comorbidities index, BMI: Body Mass Index, NMS: The New Mobility Score.

**Figure 3:**
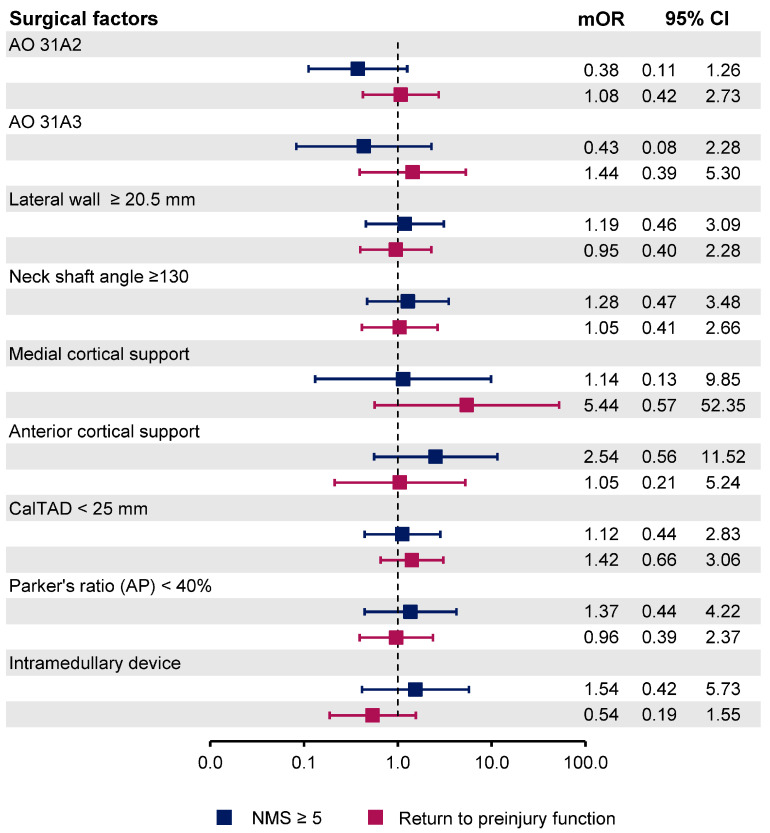
Coefficient plot presenting the odds ratio of each surgical related parameter regarding two different endpoints (NMS ≥ 5 and the ability to return to preinjury functional status). AO: AO/OTA classification, CalTAD: Calcar reference Tip Apex Distance, NMS: The New Mobility Score.

In univariable logistic regression, the patient’s age, pre-injury NMS, preoperative hemoglobin level, CCI, BMI, length of stay, and ambulatory status at discharge time were statistically significant predictors (Table 3). Of the 18 predictors included for the multivariable logistic regression analysis, four were identified as independent predictors of postoperative ambulatory status at one year. Pre-injury NMS ≥ 5 was the predictor with the largest effect size (mOR 52.72, 95%CI 5.19–535.77, *p* = 0.001). Other influential predictors were preoperative hemoglobin level ≥ 10 g/dL (mOR: 3.26, 95% CI 1.11–9.57, *p* = 0.031), BMI < 23 kg/m^2^ (mOR 3.14, 95% CI 1.21–8.13, *p* = 0.018), and the ambulatory status of patients at discharge time with NMS at least 2 (mOR 8.50, 95% CI 3.33–21.70, *p* < 0.001). None of the surgical-related factors have a statistically significant effect on postoperative functional status (Table 3). In multivariable logistic regression analysis for factors associated with the ability to return to preinjury functional status, only patient’s age (mOR 2.34, 95% CI 1.11–4.93, *p* = 0.025) and ambulatory status at discharge time with NMS at least 2 (mOR 6.09, 95% CI 2.75–14.32, *p* < 0.001) were identified as independent predictors (Table 4). The varying effect of each predictor between two different endpoints was summarized in regression coefficient plots (Figure 2 and Figure 3).

## 4. Discussion

In this study, it was revealed that the effect of surgical-related factors on the postoperative ambulatory status at one year was outweighed by parameters that reflect the patients’ baseline clinical and functional status, such as the pre-injury NMS, which was the strongest predictive factors. The others clinical parameters identified were the NMS at hospital discharge, BMI, and preoperative Hb level. Only patient’s age and functional status at hospital discharge were identified as an independent predictor for returning to preinjury functional status.

For patients with an intertrochanteric fracture who underwent surgery, their baseline functional status both before the injury and at discharge were identified as important predictors for their postoperative ambulatory status, which was in consistent with previous evidence. The better the pre-injury ambulatory status, the better the functional outcome would be postoperatively [28]. Patients with lower BMI were more likely to ambulate at one year. We hypothesized that patients with higher BMI might be less physically active and have insufficient caloric expenditure, which leads to the continuous deterioration of their ambulatory status [29]. Therefore, an early rehabilitation intervention to promote sufficient physical activity and nutritional optimization to achieve a balance caloric intake and expenditure should be incorporated into a perioperative protocol for older patients with intertrochanteric fractures.

Admission hemoglobin concentration has been extensively studied and was consistently reported as prognostic factors for functional outcomes and mortality in patients with hip fracture [21]. Based on our results, we recommended that orthopedists should maintain the preoperative Hb level greater than 10 g/dL to improve the postoperative functional recovery [13]. Although an early surgery did not result in a significant improvement of the functional outcome in this study, the surgical operation should still be performed as soon as possible, or within 48 h, in this domain of patients, as it was proven to reduce the length of hospital stay, postoperative complications, and mortality [30]. Ambulatory status at discharge time positively affected the 1-year postoperative outcome of the patients, similar to the previous study [28]. 

In our study, only less than one-third of the patients could return to their preinjury functional status at one year, similar to the numbers reported by previous studies, from 29% to 39% [2,31]. Interestingly, although a good baseline functional status could strongly predict an acceptable postoperative ambulatory status, it could not predict the ability of the patients to return to their preinjury functional status as the ability to return to preinjury functional status was independent of the patients’ baseline functional status. In contrast, only functional status at discharge were significantly associated with both study endpoints. This finding emphasizes the importance of adequate postoperative rehabilitation, which positively influences the patient’s long-term functional outcome [28]. Patients aged less than 80 years were more likely to return to their preinjury functional status. Although the statistical significance of this factor was identified only for the secondary endpoint, the direction and magnitude of the association were somewhat similar for the primary endpoint. Thus, it might be reasonable to conclude that age was associated with functional recovery, which was concordance with a previous study [32]. Based on our findings, we suggest that an adequate postoperative rehabilitation to enable the patients to regain at least an NMS of 2, or self-ambulation with a walking aid, is encouraged before hospital discharge.

Contrary to our prior hypothesis, surgical-related parameters were not significantly associated with the postoperative functional outcome at one year, either NMS ≥ 5 or the ability to return to preinjury functional status. However, the absence of statistical evidence should not be construed as the absence of the prognostic ability. Since orthopedic surgeons would attempt to obtain the optimal quality of surgical reduction and fixation in every operation, the effect of these surgical parameters was obscured and might require a larger sample size to identify the statistical significance. Based on the direction and the magnitude of the effect estimates, some surgical-related parameters should not be overlooked, such as the figure configuration. The AO/OTA 31A2 and 31A3 fracture configurations reduced the odds of postoperative ambulation by 0.38 and 0.43, respectively. Intertrochanteric fracture classified as AO/OTA 31A2 creates structural defect at the postero-medial zone, while AO/OTA 31A3 loses the integrity of the supero-lateral zone, which greatly reduces the stability of the intertrochanter area [18,33]. Positive to negative anterior cortical support of less than 7 mm tended to improve the outcome (mOR: 2.54, 95%CI 0.56–11.52), which might be explained according to the previous finite element analysis that shows better reduction stability with positive anterior cortical support by preventing further sliding of proximal fragment [34]. Nevertheless, the standardized surgical techniques that rely on previously reported reduction and fixation parameters should continue to be used regardless of their lack of predictive ability of the postoperative functional outcome as they have been proven to improve the biomechanical properties of the fracture fixation [7,10,18].

Although the effects of baseline clinical factors and pre-injury functional status on postoperative functional status seem to be more pronounced than surgical-related factors, orthopedic surgeons should still optimize the quality of surgery and provide each patient with effective perioperative management to improve functional recovery and promote early ambulation. During the past years, a fast-track perioperative protocol, such as an enhanced recovery after surgery (ERAS), primarily designed to be used in colorectal surgery, has been shown to provide better surgical outcomes if properly implemented. ERAS comprises three main components: preoperative nutritional optimization and education, minimally invasive intraoperative procedure with optimum fluid balance, and adequate postoperative pain control with early rehabilitation [35]. A rehabilitation program in an ERAS protocol promotes pain-free musculoskeletal function and improves overall functional recovery and postoperative ambulation [36]. Several studies had discovered the potential effectiveness of using an ERAS program in the context of orthopedic surgery [37,38]. One propensity score-matched study compared an effect of an ERAS program on patients who underwent hip and knee replacement surgery and found an enhancement in postoperative ambulation and reduction in the length of hospital stay [39]. Our findings could be used to guide the development of an ERAS program specifically designed for older patients with intertrochanteric fractures. 

Our study carried some strengths. First, this study was one of few studies to clarify the association between surgical-related and postoperative functional outcomes in patients with an intertrochanteric fracture [40]. Even though statistical significances were not identified among these surgical factors, some have potential predictability of the postoperative ambulation status based on their direction and effect size. Second, our study was able to identify the minimal acceptable NMS score of postoperative ambulation status before hospital discharge, which can be used to guide physicians to consider additional rehabilitation programs. 

The results from our study, however, should be considered in light of some limitations. First, the retrospective nature itself is subjected to several biases. Second, the exclusion of patients who passed away before the time of the interview might create an inevitable selection bias. Even though the mortality rate in our study was similar to the previous reports at approximately 15% [41], our samples might not reflect the actual underlying population. Third, using telephone interviews as the data collection method might give rise to both recall and interviewer biases. In our study, these biases were minimized by using valid assessment tools, structured interviews, and blinded interviewers [26]. Fourth, the accuracy of BMI and some other physician-estimated parameters might be questionable. However, clinicians usually perform these estimations in their practice, especially when an actual measurement was not feasible. While using this approach may affect the internal validity of the predictors, it did, however, enhance the generalizability to the real-world practice. Fifth, cognitive status, a potential influencing factor for the functional outcome, was not included in the analysis as it was not routinely evaluated or documented in our practice. Finally, the available sample size was relatively small and was not powered enough to identify the significance of some predictors. The findings of our study regarding the association between surgical-related parameters should be considered preliminary evidence, which still requires further prospective study with a larger sample size to confirm.

## 5. Conclusions

Our study emphasizes the importance of the patients’ preinjury clinical status, which outweighs the surgical-related factors in predicting postoperative functional outcomes of older patients with an intertrochanteric fracture. Pre-injury ambulatory status is the strongest independent predictor, follow by the patient’s BMI. Orthopedic surgeons should focus more attention on improving the patient’s clinical condition as well as maintaining the optimal quality of surgery. A preoperative hemoglobin level of at least 10 g/dL should be targeted. An adequate rehabilitation program should be included in the postoperative protocol to maximize the ambulatory capability before discharge and ensure a good 1-year postoperative ambulation, allowing patients to return to their previous functional status.

## Figures and Tables

**Table 1:** Demographic data and clinical factors in 209 patients comparing between good and poor functional outcome at one year.

Variable	Total (209)	NMS ≥ 5 (149, 71.3%)	NMS < 5 (60, 28.7%)	*p*-Value
Age (mean ± SD) years	82 ± 7	81 ± 7	84 ± 6	0.008
Age ≥ 80 years	75 (35.9%)	61 (40.9%)	14 (23.3%)	0.017
Male gender (n.%)	55 (26.3%)	42 (28.2%)	13 (21.7%)	0.388
Pre-fracture NMS ^†^ (median, IQR)	9 (6, 9)	9 (7, 9)	6 (4, 9)	<0.001
Pre-fracture NMS ≥ 5	192 (91.9%)	148 (99.3%)	44 (73.3%)	<0.001
Hb ^‡^(mean ± SD) g/dL	10.6 ± 1.7	10.7 ± 1.6	10.2 ± 1.7	0.022
Hb ≥ 10 g/dL	62 (29.7%)	54 (36.2%)	8 (13.3%)	0.001
CCI ^§^(median, IQR)	4 (4, 5)	4 (4, 5)	5 (4, 6)	<0.001
CCI < 5	114 (54.6%)	90 (60.4%)	24 (40.0%)	0.009
BMI ^¶^(mean ± SD) kg/m^2^	21.7 ± 3.6	21.4 ± 3.6	22.5 ± 3.9	0.062
BMI < 23 kg/m^2^	149 (71.3%)	114 (76.5%)	35 (58.3%)	0.011
Albumin (mean ± SD) mg/L	3.6 ± 0.5	3.7 ± 0.5	3.6 ± 0.4	0.117
Albumin ≥ 3 mg/L	183 (87.6%)	129 (86.6%)	54 (90.0%)	0.645
Time to surgery (median, IQR) (d)	5 (3, 8)	4 (2, 7)	5 (3, 8)	0.066
Time to surgery < 3 days	80 (38.3%)	63 (42.3%)	17 (28.3%)	0.083
Length of stay (median, IQR) (d)	12 (9, 15)	11 (8, 14)	14 (11, 18)	<0.001
Length of stay < 14 days	148 (70.8%)	114 (75.5%)	34 (56.7%)	0.007
NMS ^†^ at discharge (median, IQR)	3 (1, 4)	3 (2, 5)	1 (0, 2)	<0.001
NMS at discharge ≥ 2	109 (52.2%)	96 (64.4%)	13 (21.7%)	<0.001

^†^ The New Mobility Scores, ^‡^ Hemoglobin, ^§^ Charlson’s comorbidity index, ^¶^ Body Mass Index.

**Table 2:** Surgical-related factors in 209 patients comparing between good and poor functional outcome at one year.

Variable	Total (209)	NMS ≥ 5 (149, 71.3%)	NMS < 5 (60, 28.7%)	*p*-Value
Fracture classification (*n*, %)				
31A1	57 (27.3%)	47 (31.5%)	10 (16.7%)	
31A2	116 (55.5%)	77 (51.7%)	39 (65.0%)	
31A3	36 (17.2%)	25 (16.8%)	11 (18.3%)	0.086
Lateral wall thickness (mean ± SD) mm	21.3 ± 6.6	21.7 ± 6.8	20.5 ± 5.9	0.220
Lateral wall thickness ≥ 20.5 mm	108 (51.7%)	79 (53.0%)	29 (48.3%)	0.545
Neck shaft angle (mean ± SD) °	134.9 ± 8.4	135.3 ± 7.8	133.9 ± 9.8	0.256
Neck shaft angle ≥ 130°	153 (73.2%)	116 (77.9%)	37 (61.7%)	0.024
Medial cortical support (mean ± SD) mm	(+) 0.7 ± 3.7	(+) 0.7 ± 3.5	(+) 0.8 ± 4.2	0.916
Negative medial cortical support < 6 mm	200 (95.7%)	144 (96.6%)	56 (93.3%)	0.282
Anterior cortical support (mean ± SD) mm	(−) 1.2 ± 4.1	(−) 1.1 ± 3.7	(−) 1.4 ± 4.9	0.646
Negative anterior cortical support < 7 mm	193 (92.3%)	140 (94.0%)	53 (88.3)	0.247
CalTAD ^†^ (mean ± SD) mm	26.6 ± 5.9	26.6 ± 5.9	26.5 ± 6.1	0.957
CalTAD < 25 mm	89 (42.6%)	66 (44.3%)	23 (38.3%)	0.445
Parker’s ratio (AP) (mean ± SD) %	47.8 ± 8.2	47.4 ± 8.0	48.8 ± 8.5	0.257
Parker’s ratio (AP) < 40%	43 (20.6%)	34 (22.8%)	9 (15.0%)	0.258
Fixation implant				
Extramedullary device	42 (20.1%)	32 (21.5%)	10 (16.7%)	
Intramedullary device	167 (79.9%)	117 (78.5%)	50 (83.3%)	0.567

^†^ Calcar reference tip-apex distance.

**Table 3:** Logistic regression analysis of prognostic factors of regaining good 1-year postoperative ambulatory status (NMS ≥ 5).

Variable	Univariable			Multivariable		
	uOR	95% CI	*p*-Value	mOR	95% CI	*p*-Value
Age ≥ 80	2.28	1.15–4.50	0.018	2.50	0.95–6.58	0.064
Male gender	1.42	0.70–2.89	0.334	1.79	0.62–5.14	0.279
Pre-fracture NMS ^†^ ≥ 5	53.82	6.94–417.27	<0.001	52.72	5.19–535.77	0.001
Hb ^‡^ ≥ 10 g/dL	3.69	1.63–8.35	0.002	3.26	1.11–9.57	0.031
CCI ^§^ < 5	2.29	1.24–4.22	0.008	2.02	0.85–4.84	0.113
BMI ^¶^ < 23 kg/m^2^	2.33	1.23–4.40	0.009	3.14	1.21–8.13	0.018
Albumin ≥ 3 mg/L	0.72	0.27–1.88	0.499	0.50	0.14–1.79	0.285
Fracture classification						
31A1	1.00	Reference		1.00	Reference	
31A2	0.42	0.19–0.92	0.030	0.38	0.11–1.26	0.113
31A3	0.48	0.18–1.29	0.148	0.43	0.08–2.28	0.324
Lateral wall thickness ≥ 20.5 mm	1.21	0.66–2.20	0.540	1.19	0.46–3.09	0.722
Neck shaft angle ≥130°	2.19	1.14–4.18	0.018	1.28	0.47–3.48	0.628
Negative medial cortical support < 6 mm	2.06	0.53–7.94	0.295	1.14	0.13–9.85	0.907
Negative anterior cortical support < 7 mm	2.05	0.73–5.80	0.174	2.54	0.56–11.52	0.228
CalTAD ^††^ < 25 mm	1.28	0.69–2.36	0.431	1.12	0.44–2.83	0.813
Parker’s ratio (AP) < 40%	1.68	0.75–3.75	0.209	1.37	0.44–4.22	0.585
Fixation implant						
Extramedullary device	1.00	Reference		1.00	Reference	
Intramedullary device	0.73	0.33–1.60	0.434	1.54	0.42–5.73	0.516
Time to surgery < 3 days	1.85	0.97–3.55	0.062	0.55	0.19–1.55	0.256
Length of stay < 14 days	2.30	1.23–4.30	0.009	2.42	0.91–6.42	0.077
NMS ^†^ at discharge ≥ 2	6.55	3.25–13.18	<0.001	8.50	3.33–21.70	<0.001

^†^ The New Mobility Scores, ^‡^ Hemoglobin, ^§^ Charlson’s comorbidity index, ^¶^ Body Mass Index, ^††^ Calcar reference tip-apex distance.

**Table 4:** Logistic regression analysis of prognosis factors of returning to preinjury functional status at one year.

Variable	Univariable			Multivariable		
	uOR	95% CI	*p*-Value	mOR	95% CI	*p*-Value
Age ≤ 80	2.23	1.18–4.20	0.013	2.34	1.11–4.93	0.025
Male gender	0.97	0.48–1.97	0.940	0.86	0.37–2.01	0.727
Pre-fracture NMS ^†^ ≥ 5	1.14	0.36–3.67	0.821	0.51	0.12–2.12	0.356
Hb ^‡^ ≥ 10 g/dL	0.88	0.44–1.76	0.725	0.66	0.29–1.50	0.320
CCI ^§^ < 5	1.44	0.76–2.70	0.262	1.17	0.54–2.53	0.689
BMI ^¶^ < 23 kg/m^2^	1.21	0.60–2.43	0.600	1.36	0.57–3.24	0.490
Albumin ≥ 3 mg/L	0.94	0.37–2.37	0.892	0.51	0.16–1.63	0.255
Fracture classification						
31A1	1.00	Reference		1.00	Reference	
31A2	0.82	0.40–1.67	0.577	1.08	0.42–2.73	0.878
31A3	0.99	0.39–2.50	0.976	1.44	0.39–5.30	0.583
Lateral wall thickness ≥ 20.5 mm	1.12	0.60–2.08	0.729	0.95	0.40–2.28	0.911
Neck shaft angle ≥130°	1.60	0.76–3.40	0.218	1.05	0.41–2.66	0.919
Negative medial cortical support < 5 mm	5.26	0.68–41.01	0.113	5.44	0.57–52.35	0.142
Negative anterior cortical support < 7 mm	1.56	0.43–5.69	0.503	1.05	0.21–5.04	0.948
CalTAD ^††^ < 25 mm	1.36	0.73–2.53	0.338	1.42	0.66–3.06	0.374
Parker’s ratio (AP) < 40%	1.14	0.54–2.43	0.728	0.96	0.39–2.37	0.935
Fixation implant						
Extramedullary device	1.00	Reference		1.00	Reference	
Intramedullary device	0.73	0.35–1.53	0.398	0.54	0.19–1.55	0.253
Time to surgery < 3 days	1.28	0.68–2.40	0.449	0.74	0.33–1.69	0.482
Length of stay < 14 days	1.86	0.89–3.91	0.101	2.13	0.90–5.03	0.086
NMS ^†^ at discharge ≥ 2	6.09	2.86–12.99	<0.001	6.27	2.75–14.32	<0.001

^†^ The New Mobility Scores, ^‡^ Hemoglobin, ^§^ Charlson’s comorbidity index, ^¶^ Body Mass Index, ^††^ Calcar reference tip-apex distance.

## Data Availability

The datasets used and/or analysed during the current study are available from the corresponding author on reasonable request. The data are not publicly available due to their containing information that could compromise the privacy of research participants.
